# A novel workflow combining plaque imaging, plaque and plasma proteomics identifies biomarkers of human coronary atherosclerotic plaque disruption

**DOI:** 10.1186/s12014-017-9157-x

**Published:** 2017-06-19

**Authors:** Regent Lee, Roman Fischer, Philip D. Charles, David Adlam, Alessandro Valli, Katalin Di Gleria, Rajesh K. Kharbanda, Robin P. Choudhury, Charalambos Antoniades, Benedikt M. Kessler, Keith M. Channon

**Affiliations:** 1 Division of Cardiovascular Medicine, University of Oxford, Oxford, UK; 2 Acute Vascular Imaging Centre, Radcliffe Department of Medicine, University of Oxford, Oxford, UK; 3 Target Discovery Institute, Nuffield Department of Medicine, University of Oxford, Oxford, UK; 4 National Institute for Health Research (NIHR) Oxford Biomedical Research Centre, John Radcliffe Hospital, Oxford University Hospitals NHS Foundation Trust, Oxford, UK

**Keywords:** Coronary atherosclerosis, Plaque rupture, Proteomics, Biomarkers

## Abstract

**Background:**

Atherosclerotic plaque rupture is the culprit event which underpins most acute vascular syndromes such as acute myocardial infarction. Novel biomarkers of plaque rupture could improve biological understanding and clinical management of patients presenting with possible acute vascular syndromes but such biomarker(s) remain elusive. Investigation of biomarkers in the context of de novo plaque rupture in humans is confounded by the inability to attribute the plaque rupture as the source of biomarker release, as plaque ruptures are typically associated with prompt down-stream events of myocardial necrosis and systemic inflammation.

**Methods:**

We developed a novel approach to identify potential biomarkers of plaque rupture by integrating plaque imaging, using optical coherence tomography, with both plaque and plasma proteomic analysis in a human model of angioplasty-induced plaque disruption.

**Results:**

We compared two pairs of coronary plaque debris, captured by a FilterWire Device, and their corresponding control samples and found matrix metalloproteinase 9 (MMP9) to be significantly enriched in plaque. Plaque contents, as defined by optical coherence tomography, affect the systemic changes of MMP9. Disruption of lipid-rich plaque led to prompt elevation of plasma MMP9, whereas disruption of non-lipid-rich plaque resulted in delayed elevation of plasma MMP9. Systemic MMP9 elevation is independent of the associated myocardial necrosis and systemic inflammation (measured by Troponin I and C-reactive protein, respectively). This information guided the selection of a subset of subjects of for further label free proteomics analysis by liquid chromatography tandem mass spectrometry (LC–MS/MS). We discovered five novel, plaque-enriched proteins (lipopolysaccharide binding protein, Annexin A5, eukaryotic translocation initiation factor, syntaxin 11, cytochrome B5 reductase 3) to be significantly elevated in systemic circulation at 5 min after plaque disruption.

**Conclusion:**

This novel approach for biomarker discovery in human coronary artery plaque disruption can identify new biomarkers related to human coronary artery plaque composition and disruption.

**Electronic supplementary material:**

The online version of this article (doi:10.1186/s12014-017-9157-x) contains supplementary material, which is available to authorized users.

## Background

Atherosclerotic plaque rupture is the culprit event which underpins a majority of acute vascular syndromes such as acute myocardial infarction [1], yet biomarkers of plaque rupture remain elusive [2, 3]. Novel biomarkers of plaque rupture could improve clinical management of patients presenting with possible acute vascular syndromes, and give new insights to the pathophysiological process causing plaque rupture, and the local and systemic consequences.

One of the challenges in the investigations of plaque rupture in humans lies in the difficulty to establish the time course of a de novo plaque rupture event. In this regard, plaque disruption by percutaneous coronary intervention (PCI) induces controlled plaque rupture in humans and allows profiling of biomarker changes timed precisely to a plaque event [4–6]. Furthermore, intra-arterial imaging of plaque in vivo using optical coherence tomography (OCT) can define plaque composition, providing additional information to improve the specificity of biomarker discovery [6–9].

However, even in such controlled settings, characterisation of biomarker of plaque disruption are further complicated by the presence of downstream effects such as myocardial necrosis and systemic inflammation after PCI [7, 10, 11], as these downstream events also lead to changes in cardiac enzymes (cTnI) and c-reactive protein (CRP) and can confound the identification of other circulating biomarkers that are specifically associated with plaque disruption.

In this study, we applied a novel discovery workflow to the human model of PCI-induced plaque disruption. We hypothesised that by combining the information from coronary plaque imaging analysis, proteomics analysis of coronary plaque material, and proteomics analyses of serial plasma samples collected before and after plaque disruption, this would enable identification plaque proteins acutely elevated in systemic circulation that are specific markers of human coronary artery plaque disruption.

## Methods

### Model of human plaque disruption in vivo

In a prospective study at the Oxford Radcliffe Hospital (ORH), Oxford, UK, patients between the age of 30–90 who were referred for non-emergency coronary angiography with the view to proceed to PCI were invited to take part. Patients who underwent diagnostic angiography without stenting served as control subjects in this experimental model as the steps involved in diagnostic angiography (arterial needle puncture/catheterisation, intra-arterial passage of guidewires, injection of contrast and acquisition of X-ray images) were the same as PCI except deployment of stents. We excluded patients who were haemodynamically unstable, or when there was a known history of renal impairment or anaemia. Patients undergoing left main coronary artery angioplasty were also excluded. The study was approved by the Oxford Regional Ethics Committee (Ethics Ref: 08/H0603/41) and complies with the Helsinki Declaration. Written informed consent was obtained from every participant.

### Characterisation of plaque morphology by optical coherence tomography (OCT)

OCT images were acquired using the Lightlab system (St. Jude Medical, Minnesota, USA) prior to disruption of plaque by stent. Image analyses were performed by assessors who were blinded to the clinical status and other biomarker measurements of the patient. The culprit lesion was categorised into two subtypes (lipid-rich and non-lipid rich) using the pre-defined scoring system. (Full details on OCT image acquisition and analysis please in Additional file 1.) Discordance between observers were resolved by taking a consensus reading. A binary score was then assigned to each culprit lesion for comparison with other variables.

### Sampling of coronary plaque material

**Fig. 1:**
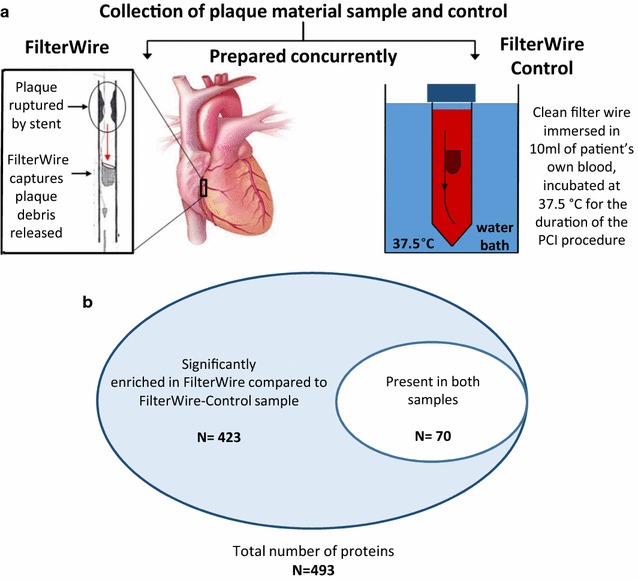
Establishing a library of protein that are present in coronary plaque debris, captured by the FilterWire device. **a** In order to identify proteins acutely released into circulation from a disrupted plaque, we first established a library of plaque proteins by untargeted proteomic analysis of plaque debris captured by a distal protection device with pore size of 110 μm. (FilterWire, Boston Scientific), positioned in the coronary artery downstream from the plaque during PCI. Concurrently, a second FilterWire was incubated at 37.5 °C ex vivo in heparinised blood from the same patient (obtained at the beginning of the procedure, before angioplasty), for the same duration as the PCI procedure. This separate FW served as a control sample for the one deployed in the coronary artery in the same patient, in order to identify the non-specific blood proteins which may adhere to the FW during the PCI process, other than plaque debris. **b** These paired samples were analysed using the proteomics workflow as described in the manuscript. 423 proteins were significantly enriched in FilterWire compared to FilterWire-Control and considered to be plaque specific proteins

To obtain coronary plaque material released after its disruption, a FilterWire device (FW, Boston Scientific, Massachusetts, USA) was deployed downstream of the lesion in a standard manner. The umbrella-like polyurethane mesh contains 110 µm pores to allow passage of blood components while trapping the plaque material released during angioplasty. To assess if blood components (other than coronary plaque material) would also adhere to the FW during the PCI procedure, therefore giving rise to artefactual signals unrelated to coronary plaque material, a paired FW device was prepared concurrently as the matched control sample in the following manner: after the diagnostic angiography confirmed suitability for stenting with FW distal protection, an additional 10 ml of arterial blood was aspirated from the coronary guide catheter and transferred into a 15 ml Falcon tube. A FW was then positioned downstream of the coronary lesion followed by the PCI procedure. Concurrently, an unused FW was placed in the Falcon tube containing the individual’s blood. This was then immediately transferred to a water bath and incubated at 37.5 °C for the duration of the stenting procedure (Fig. 1). At the end of the PCI procedure , both FW (one containing plaque material, and one from ex vivo incubation) were retrieved and immediately frozen and stored at −80 °C for subsequent analysis.

### Serial plasma sampling

Blood samples were collected from coronary artery and peripheral vein at specific time points after baseline. Standard guiding catheters were used for blood sampling from proximal coronary artery during the PCI procedure (at baseline and at 5 min). Peripheral venous blood samples were obtained from either indwelling venous catheters or fresh venepuncture (at baseline, 1, 6, 18 h). The same time points applied to diagnostic angiography patients except for stent placement, and the beginning of procedure was used as the baseline. Blood samples were prepared without delay in the cardiac catheter laboratory at room temperature to obtain platelet-poor plasma with the following protocol: BD Vacutainer^®^ containing K_2_EDTA was used for the first spin of blood (12 min × 1300 g). The plasma portion was then transferred to a second BD Vacutainer^®^ without additive for the second spin (15 min × 2500 g). Confirmation of platelet-poor plasma was confirmed by platelet counts [12]. Plasma aliquots were stored at −80 °C for subsequent analysis.

### Candidate marker analysis

Measurement of plasma CRP, cTnI (both by high sensitivity assays), and blood cholesterol profiles were performed as clinical blood tests by the biochemistry at the Oxford Radcliffe Hospital. Other candidate markers were measured using commercially available bead based immune-sorbent assays according to the manufacturer’s instructions. (MMP9: HCVD1-67AK, Merck Millipore, USA; LBP: Mybiosource, MBS721952, USA). Intra- and inter-plate variations (%CV) for these assays were less than 10%.

### Statistical analysis for candidate markers

Dependent on the normality of sample distribution, comparisons between two groups were performed by either parametric or non-parametric t-tests. Log transformation of data was performed where appropriate. Analysis of variance (ANOVA) was performed for comparison between 3 or more groups or to test the effects of repeated measures. Area under curve (AUC) of the time course response for each candidate marker was calculated. These analyses were performed using Graphpad Prism (Version 5.0, San Diego California USA).

Data with a Gaussian distribution are presented as mean ± standard error of mean; parametric data are presented as median and 25–75 percentile in box plots. Area under curve (AUC) can be seen as relative measure of abundance in release over a period, which allows comparisons across samples/patients. AUC over the time course of each biomarker was calculated using the “area under curve” analysis in GraphPad Prism. As there is a broad physiological range of baseline MMP9 concentration between individuals, the fold change of MMP9 at each time point (against baseline) was used for the AUC calculation. In contrast, the baseline levels of CRP and Troponin are fairly consistent between individuals (hence their role as diagnostic tests). The AUC for CRP and Troponin are therefore calculated using the absolute concentration measured at each time point.

Correlation between MMP9 and known injury and inflammation response biomarkers (Troponin and CRP) was assessed by Spearman’s rank correlation coefficient (Spearman’s rho) across individuals to capture any monotonic relationship (linear or otherwise). Significance was determined by converting the Fisher transformation of the rho values to z-scores, thus yielding corresponding *P* values by assuming the z-scores to follow a standard normal distribution (calculations performed in R).

### Sample size consideration for proteomics analysis

Sample size consideration in comparative proteomics research, whereby samples are compared according to a disease condition (such as before and after plaque disruption) to screen for up or down regulation of “novel/unknown” molecular signatures, is different from the traditional power calculation approach for “known” candidate markers [13]. Assuming the combined biological and technical variance of 75% between samples, the sample size of 10 in each arm of the study will be sufficient to detect a 1.9 fold change (up or down regulation) in a given protein/metabolite with 80% power and α of 0.05 [14].

### Untargeted proteomic analysis of coronary plaque and plasma samples

For the discovery analysis by shot gun proteomics, we compared plasma samples taken at *baseline* and at *5* *min* to capture the earliest changes detectable in circulation. Changes observed at this early time point are most likely due to acute liberation of pre-existing proteins into circulation. Plasma samples for each group were pooled for subsequent analysis, as pooling gave the advantage of reducing the intrinsic biological variation observed between samples, making the substantive features easier to detect, and reduced the total number of samples analysed and reduce the overall requirement of computational power for the analysis and the constraints imposed by instrument throughput and cost considerations [15, 16]. Samples were prepared using in-solution digestion with trypsin. Mass spectra of these samples were acquired using LC–MS/MS (LTQ-orbitrap Velos, Thermo Fisher Scientific). For detailed description of the sample preparation and mass spectra acquisition, please see Additional file 1.

### Label-free relative quantitation of proteins

For label-free protein quantitation, raw data were first imported to Progenesis LC–MS and processed through the default workflow. Proteins were grouped and only protein features which were identified by two or more tryptic peptides were included for identification. Statistical comparisons were made in the following experimental groups: FW versus FW-Controls, plasma no plaque disruption (0 vs. 5 min), plasma plaque disruption (0 vs. 5 min). For the comparison between FW and FW-controls, normalisation of protein abundance was not performed because of the vastly low abundance of protein in the FW-Controls. For the comparison between paired plasma samples, normalisation was performed against all peptides as there were comparable intensity of mass spectra in paired samples. *P* values for comparison were calculated by the Progenesis LC–MS software, based on paired *t* tests (two sample ANOVA), and the value of <0.05 was considered the cut-off point for selection of a putative protein feature for further examination.

Only peptides identified with a false discovery rate of 1% and with ion score of >20 were accepted for further analysis. False discovery rate (FDR) of the protein identification was further performed by uploading the converted MGF files to the Central Proteomics Facilities Pipeline (CPFP, version 1.3.0 http://www.proteomics.ox.ac.uk/), which combined the searches against the human databases using three different search engines (Mascot, OMSSA, and X!TANDEM k-score) [17]. The FDR for each protein was cross referenced between CPFP output file and Progenesis LC–MS data. Of these proteins which had a *P* value less than 0.05 on semi-quantitative analysis, only those which fulfilled a FDR of less than 1% according to CPFP analysis were considered for further analysis.

### Ingenuity Pathway Analysis (IPA) of proteomics datasets

The complete plasma protein dataset for the individual experimental groups were further exported from Progenesis LC–MS. For each dataset, the following values were assigned as an observation in IPA: protein accession number, ANOVA *P* value, fold change at 5 min (compared to 0 min). Canonical pathways implicated by the plasma proteins in the sample groups were assessed using the over-representation technique in IPA. This analysis provided the significance *P* value based on likelihood of representation and the percentage representation of proteins detected in the dataset. Benjamini–Hochberg correction for multiple testing was applied to the *P* values.

## Results

**Fig. 2:**
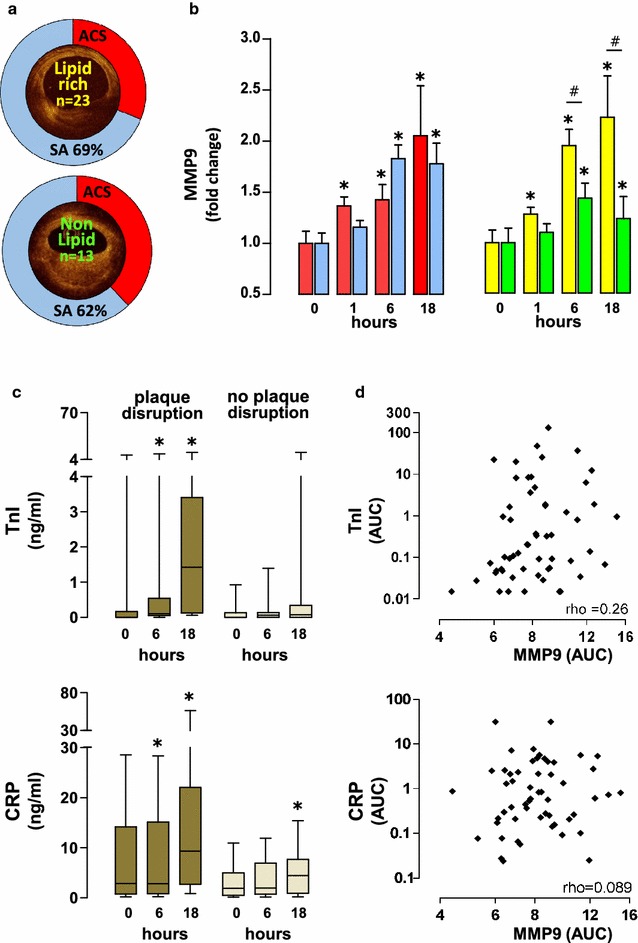
Plaque composition affects systemic elevation of MMP9 after its disruption, which is independent of the myocardial injury and systemic inflammatory response. Coronary artery plaques were imaged using optical coherence tomography (OCT) prior to plaque disruption, in 36 patients undergoing percutaneous coronary intervention (PCI), comparing with subjects who underwent diagnostic coronary angiography without PCI (Dx). These plaques were classified as either lipid-rich (*yellow*, n = 23) or non-lipid-rich plaques (*green*, n = 13) based on OCT characteristics. Lipid-rich and non-lipid rich plaques were observed in patients with either acute coronary syndrome (ACS-*red*, n = 21) or stable angina (SA-*blue*, n = 37) (**a**). More prompt elevation in plasma MMP9 was observed in patients who presented with ACS (**b**, *red bars*) at 1 h after plaque disruption (compared to those who presented to SA, *blue bars*), but there was no difference in the peak MMP9 levels observed after 6 h. In contrast, disruption of lipid-rich plaques (**b**, *yellow bars*) led to more prompt elevations of MMP9 and higher peak level after 6 h. To test for possible confounding effects of myocardial injury and systemic inflammatory response after plaque disruption, we measured circulating troponin I (TnI) and c-reactive protein (CRP) in all participants (PCI n = 58; Dx n = 23). Myocardial injury was only observed in the group with plaque disruption. Modest elevation in CRP was observed in both groups, with or without plaque disruption (**c**). Calculation of area under curve (AUC) revealed no correlation between MMP9 and either TnI or CRP (**d**) during this time course. These observations indicate that MMP9 release is specific to the upstream event of plaque disruption, and independent of the downstream myocardial injury and systemic inflammation as a result of the procedure. “*” denotes significant statistical comparison against the baseline measurement (paired comparison); “#” denotes significant statistical comparison between the two groups (lipid-rich vs. non-lipid-rich)

We imaged coronary artery plaques, using optical coherence tomography (OCT) prior to plaque disruption, in 36 patients undergoing percutaneous coronary intervention (PCI). These plaques were classified as either lipid-rich (n = 23) or non-lipid-rich (n = 13) plaques based on OCT characteristics. Lipid-rich and non-lipid-rich plaques were observed in patients who presented with either acute coronary syndrome (ACS) or stable angina (SA) (Fig. 2a).

We first analysed plaque debris collected “downstream” from the coronary artery plaque during PCI, using a FilterWire device. The FW and the FW-Control were processed concurrently for proteomics analyses. We identified a total of 493 proteins from these FW and FW-control samples. Of these, 423 proteins were derived from the plaque debris as they were significantly enriched (*P* < 0.05) in the FW samples as compared to the paired FW-Control (Fig. 1). For the complete list please see Additional file 2. Matrix metalloproteinase 9 (MMP9) was one of the proteins significantly enriched in the plaque debris. Given the known involvement of MMP9 in vulnerable plaques [18], we selected MMP9 as a surrogate marker of the response to human coronary artery plaque disruption.

To test whether putative biomarkers of human coronary plaque disruption are also detectable in the peripheral circulation, we next measured plasma MMP9 levels after plaque disruption, in relation to clinical status [ACS (n = 21) vs. SA (n = 37)] and plaque morphology [lipid-rich (n = 23) vs. non-lipid-rich (n = 13)]. More prompt elevation in plasma MMP9 was observed in patients who presented with ACS (fold change 1.37 ± 0.08, *P* < 0.05 against baseline) at 1 h after plaque disruption (as compared to those who presented to SA with fold change of 1.16 ± 0.06). By 18 h after plaque disruption, both ACS and SA patients showed similar fold changes (ACS: 1.98 ± 0.46; SA: 1.79 ± 0.16). In contrast, disruption of lipid-rich plaques led to more prompt elevations of MMP9 (fold change 1.28 ± 0.07 at 1 h, *P* < 0.05 against baseline) and higher peak level after 6 h (fold change 1.96 ± 0.16) as compared to non-lipid-rich plaques (fold change 1.1 ± 0.08 at 1 h, and 1.44 ± 0.15 at 6 h) (Fig. 2b). There was no gender difference in the MMP9 levels observed (Data not shown). Taken together, these observations suggest plaque composition determines biomarker release after plaque disruption, providing the basis for novel biomarker discovery associated with plaque contents.

To test for possible confounding effects of myocardial injury and systemic inflammatory response after plaque disruption, we measured circulating troponin I (TnI) and C-reactive protein (CRP) in all participants (PCI n = 58; Dx n = 23, Table 1). Myocardial injury was observed only in the group with plaque disruption, after 6 h. Modest elevation in CRP was observed in both groups, with or without plaque disruption (Fig. 2c). Quantification of area under curve (AUC) values were consistent with a null hypothesis of no correlation between MMP9 and either TnI (*P* = 0.91) or CRP (*P* = ~1) during this time course (Fig. 2d). These observations indicate that MMP9 release is specific to the upstream event of plaque disruption, and independent of the downstream myocardial injury and systemic inflammation as a result of the procedure.

**Table 1:** Characteristics of study subjects included for overall analysis

	PCI	Diagnostic angiography
Number (male)	58 (46)	23 (16)
Age [years (SD)]	66 (11)	68 (10)
Acute coronary syndrome [n (%)]	21 (36)	13 (57)
Smoking status [n (%)]		
Current smoker	16 (28)	3 (13)
Ex-smoker >1 month	25 (43)	13 (57)
Never smoked	17 (29)	7 (30)
Past history of IHD [n (%)]		
MI/ACS	22 (38)	7 (30)
Stable angina	15 (26)	7 (30)
Hypertension [n (%)]	39 (67)	19 (83)
Hypercholesterolemia [n (%)]	45 (78)	13 (57)
Diabetes mellitus [n (%)]	14 (24)	4 (17)
Family history of IHD [n (%)]	29 (50)	12 (52)
Regular medication [n (%)]		
Aspirin	40 (69)	17 (74)
Thienopyridine	23 (40)	11 (48)
Statin	47 (81)	20 (87)
β-blocker	33 (57)	14 (61)
ACE inhibitor/ARB	38 (65)	19 (83)

To characterise ‘upstream’ biomarkers released immediately after plaque disruption, we used systemic MMP9 levels as a stratification tool to select a discovery cohort of patients for proteomics analyses of plasma samples collected at 5 min after plaque disruption. This cohort consisted of subjects with the highest MMP9 release (AUC) after plaque disruption, compared with a control group who underwent diagnostic angiography only. In these PCI patients with the highest MMP9_AUC_, we observed a median fold change of 1.9 at 6 h and 2.3 at 18 h. Subjects were well matched in their baseline demographics (Table 2).

**Table 2:** Demographics for the discovery cohort for proteomics analysis

	Plaque disruption	No plaque disruption
Number (male)	10 (7)	10 (5)
Age [years (SD)]	66 (8)	67 (12)
Acute coronary syndrome [n (%)]	5 (50)	6 (60)
Smoking status [n (%)]		
Current smoker	1 (10)	1 (10)
Past history of smoking (>1 month)	7 (70)	4 (40)
Never smoked	2 (20)	5 (50)
Past History of IHD [n (%)]		
MI/ACS	2 (20)	5 (50)
Stable angina	4 (40)	0 (0)
Hypertension [n (%)]	6 (60)	8 (80)
Hypercholesterolemia [n (%)]	8 (80)	6 (60)
Diabetes mellitus [n (%)]	0 (0)	0 (0)
Family history of IHD [n (%)]	6 (60)	5 (50)
Regular medication [n (%)]		
Aspirin	6 (60)	8 (80)
Thienopyridine	3 (30)	4 (30)
Statin	7 (70)	9 (70)
β-blocker	5 (50)	6 (50)
ACE inhibitor/ARB	6 (60)	7 (60)
Plasma lipid profile		
Total cholesterol [mmol/L (SD)]	3.7 (0.4)	3.8 (1.39)
HDL [mmol/L (SD)]	1.0 (0.3)	0.9 (0.3)
LDL [mmol/L (SD)]	2.0 (0.3)	2.3 (1.0)
Triglyceride [mmol/L (SD)]	1.5 (0.7)	1.5 (0.6)

**Fig. 3:**
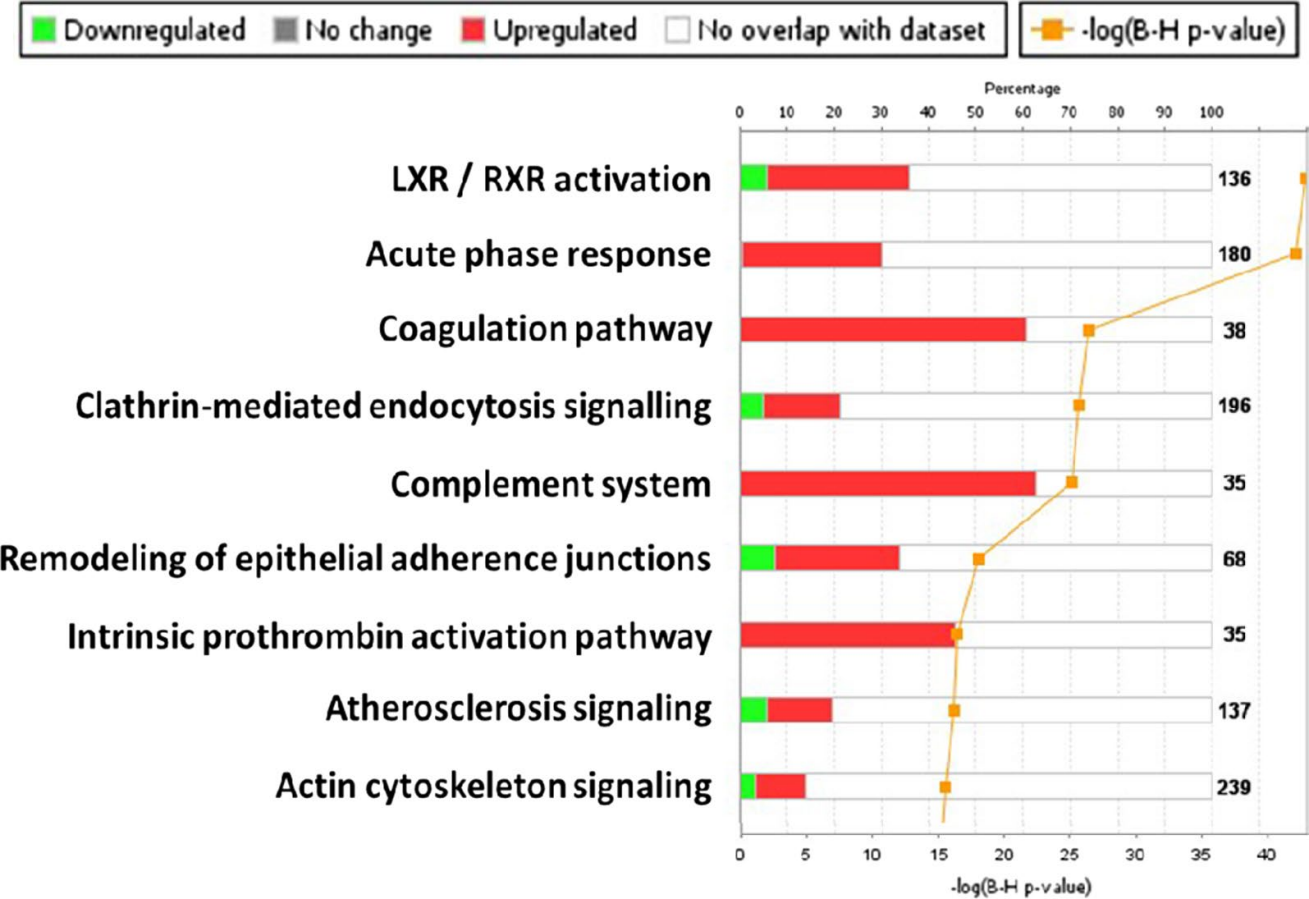
Canonical pathway analysis, using Ingenuity Pathway Analysis, highlighted the key pathways represented by these plasma proteins, with the highest representation being the Liver X receptor (LXR)/retinoic X receptor (RXR) pathway. *Top axis* the percentage representation of proteins described in the respective canonical pathway. *Bottom axis* Statistical significance of the likelihood of pathway coverage (negative log of *P* value with Benjamini–Hochberg correction for multiple testing). *Red* fraction of *bar*: up regulated proteins; *green* fraction of *bar*: down regulated proteins; *white* fraction of *bar*: no overlap with dataset

We detected 491 proteins in these plasma samples with a false discovery rate less than 1%. Canonical pathway analysis, using Ingenuity Pathway Analysis (IPA), highlighted the key pathways represented by these plasma proteins, with the highest representation being the Liver X receptor (LXR)/retinoic X receptor (RXR) pathway (Fig. 3). After exclusion of plasma proteins that were significantly changed in the control group (those who underwent diagnostic angiography only), we identified six plasma proteins that were significantly elevated (*P* < 0.05) at 5 min after plaque disruption. These were compared against the plaque protein library determined from plaque debris. Five of these proteins were also enriched in plaque debris (Table 3).

**Table 3:** Proteins that are significantly increased in systemic circulation 5 min after plaque disruption

Uniprot accession number	Protein name	Maximum fold change	ANOVA *P* value	Enriched in plaque
P08758	Annexin A5	2.41	0.0143	Yes
P18428	Lipopolysaccharide binding protein	1.75	0.0159	Yes
P63241	Eukaryotic translation initiation factors	2.28	0.0218	Yes
O75558	Syntaxin 11	3.04	0.0222	Yes
P00387	Cytochrome B5 reductase 3	3.88	0.0401	Yes
Q9UIB8	CD84	7.18	0.0486	No

We detected 491 proteins in these plasma samples with a false discovery rate of less than 1%. After exclusion of plasma proteins that were significantly changed in the control group (those who underwent diagnostic angiography only), we identified six plasma proteins that were significantly elevated (*P* < 0.05) at 5 min after plaque disruption. These were cross referenced against the plaque protein library determined from plaque debris. Five of these proteins were also present in plaque debris

Lipopolysaccharide binding protein (LBP) is one of the protein that is present in human coronary artery plaque and is significantly elevated in plasma immediately after plaque disruption. Elevation of plasma LBP levels after PCI (from 0.62 ± 0.49 to 0.74 ± 0.33 ng/ml, 1.44 times increase [95% confidence interval of 1.07–1.81]) was validated by an enzyme immunoassay in individual plasma samples, but remained unchanged in the control group who underwent diagnostic angiography without plaque disruption (Additional File 3). The significant elevation of LBP was no longer observed beyond 1 h after plaque disruption, and there was no correlation between LBP_AUC_ and TnI_AUC_ or between LBP_AUC_ and CRP_AUC_ (data not shown).

**Fig. 4:**
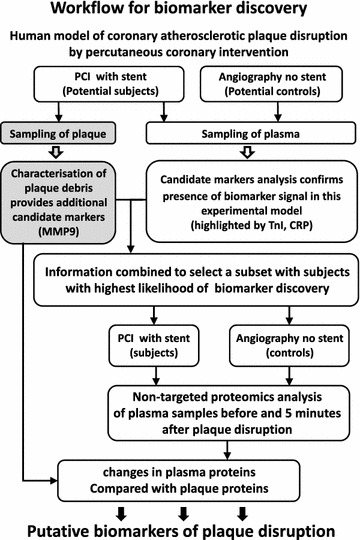
Flowchart of the proposed workflow of biomarker discovery for biomarker(s) of coronary atherosclerotic plaque rupture in humans

This novel workflow is illustrated by Fig. 4.

## Discussion

We describe a novel approach for biomarker discovery in a human model of PCI-induced coronary atherosclerotic plaque disruption. We combined plaque OCT imaging to stratify plaque composition, with discovery proteomics of both plaque debris and plasma samples, to identify and validate proteins released from human coronary artery plaques immediately after plaque disruption. This is the first time a study took such a rigorous approach of using plaque analysis to guide subsequent proteomics discovery in plasma, illustrating the potential of this approach to identify new targets and markers related to human coronary artery plaque biology.

We used OCT as a means of increasing the specificity of the study to detect proteins related to disruption of lipid-rich plaques, because these plaques (‘unstable’ or ‘vulnerable’ plaques) are the substrates for spontaneous plaque rupture that leads to acute coronary events. We observed that the pattern of biomarker release after plaque disruption (exemplified by MMP9) was independent of the clinical presentation of the patient (i.e. ACS vs. SA), but was significantly different between lipid-rich plaques compared with non-lipid plaques, demonstrating that plaque composition directly influences biomarker release after plaque disruption.

Our finding of systemic elevation in MMP9 within an hour after coronary plaque disruption is consistent with its reported role in atherosclerotic plaque destabilisation [19, 20]. That systemic MMP9 elevation after plaque disruption did not correlate with cTnI or CRP release further supports MMP9 release to be an independent feature specific to the plaque disruption.

The potential discovery utility of the integrated plaque imaging proteomic analysis approach highlights the power of combining proteomic analysis of both plaque-derived particulate debris and plasma proteins, in a rigorous and highly controlled experimental design. The plaque debris collection and analyses were conducted using a paired FilterWire device incubated in patient-derived blood ex vivo to control for proteins in circulating blood that might have otherwise adhered to the device during its deployment. This enabled identification of a highly selected list of proteins that can be specifically attributed to liberation from the plaque, after plaque disruption, and do not represent circulating plasma proteins.

We identified and validated LBP as a novel, plaque-derived biomarker of coronary atherosclerotic plaques. LBP is a secretory class 1 acute phase protein, previously associated with the response to bacterial sepsis. Various pathogen associated molecular patterns bind to LBP, and these complexes interact with Toll Like Receptor 4 (TLR4) associated pathways [21]. There is emerging literature indicating a potential role for LBP as a biomarker in atherosclerotic disease. Serum LBP has recently been shown to correlate with prevalent CAD [22] and predicted both all-cause and cardiovascular mortality [23].

Given the role of inflammatory activation by TLR4 in atherogenesis [24], our finding of LBP in human coronary artery plaques in vivo, and plaque-derived systemic release of LBP are novel findings that justify further consideration of LBP as a pathogenic factor in coronary artery disease. Indeed, release of LBP from disrupted plaque could drive the observed increase in MMP9 after plaque disruption, via TLR4 and NFκB signalling. The acute LBP elevation observed in this cohort was followed by significant subsequent increase in systemic MMP9 as described in LXR/RXR canonical pathway activation.

In addition, our workflow also identified other plasma proteins which were acutely elevated after plaque disruption that have plausible biological associations. For example, plasma Annexin A5 has previously been shown to be elevated in patient with acute myocardial infarction [25] and is acutely increased after angioplasty induced AMI [26]. Cytochrome B5 reductase 3 (CYB5R3) is an enzyme involved in the electron transfer pathway in cholesterol synthesis [27] and the elongation and desaturation of fatty acid chains [28]. High levels of circulating monounsaturated fatty acid is recently shown to associate with future cardiovascular events in a large cohorts study [29]. These observations further strengthen the validity of our approach for identifying novel targets.

Further validation by larger cohort(s) will improve the external validity of our findings and to confirm the role of these proteins as novel biomarkers. It will also be important to establish the time course profile of these proteins of plaque disruption, and the range of plasma concentration of these proteins in physiological conditions in large cohorts.

## Conclusion

We demonstrate a novel approach to discover novel biomarkers of human coronary artery plaque disruption, by combining plaque imaging with proteomic analysis of plaque-derived material, and plasma proteins. We identify the LXR/RXR pathway, and, specifically LBP, as a novel biomarker signature of human coronary artery plaques and plaque disruption. Highly-stratified proteomic analyses of plaque-derived material and plasma can identify new targets of potential biological importance in human coronary artery disease.

## Additional files

**Additional file 1.** Supplemental method and data.

**Additional file 2: Table S1.** Proteins present in the plaque debris. We identified a total of 423 proteins that were derived from the plaque debris, captured by a FilterWire positioned downstream of the disrupted plaque.

**Additional file 3.** Changes in lipopolysaccharide binding protein after plaque disruption.

## Abbreviations

ACS: acute coronary syndrome
CRP: C-reactive protein
cTnI: cardiac troponin I
CyB5R3: cytochrome B5 reductase 3
FW: FilterWire
FW-Control: FilterWire control
MMP9: matrix metalloproteinase 9
LBP: lipopolysaccharide binding protein
LC–MS: liquid chromatography mass spectrometry
LXR/RXR: liver X receptor/retinoic X receptor
OCT: optical coherence tomography
PCI: percutaneous coronary intervention
SA: stable angina
TLR4: toll like receptor 4
