# Supplementary material for: A novel workflow combining plaque imaging, plaque and plasma proteomics identifies biomarkers of human coronary atherosclerotic plaque disruption
Source: Clin Proteomics. 2017 Jun 19;14:22. doi: 10.1186/s12014-017-9157-x (PMC5477097; doi:10.1186/s12014-017-9157-x)
Supplement: Supplementary file 1 — Additional file 1. Supplemental method and data. [file 12014_2017_9157_MOESM1_ESM.docx]

# **Supplemental Methods**

**Optical coherence tomography characterisation of plaques**

#### OCT procedure

Two models of OCT console were used during the study: Lightlab M3 and C7 system (St. Jude Medical, Minnesota, USA). In both systems, the non-occlusive technique was used to achieve blood displacement by a viscous iso-osmolar contrast solution (Visipaque^TM^, GE Healthcare, UK) to allow for image acquisition. After fluoroscopy confirmation of imagewire/catheter position, image wire/catheter pull back was performed at the highest available speed during simultaneous infusion of Visipaque^TM^ (through the guiding catheter). Typically, 20ml of Visipaque^TM^ infusion was required to complete an image acquisition. The acquired raw data was stored on the OCT console for the procedure for subsequent analysis.

We did not perform OCT in cases where safety of the procedure may be compromised by the additional OCT procedure (eg small calibre vessel, anticipated long procedure etc). We did not perform OCT in patients who had a coronary angiography without PCI as this would involve the additional passage of guidewire down the coronary artery, which is not without potential risks. We were therefore not comfortable with performing the OCT step in diagnostic angiography cases.

#### Scoring system for OCT plaque morphology

For each culprit lesion, the image frame containing the minimal luminal area (MLA) was identified and selected as the index frame. Ten additional frames (5 antegrade + 5 retrograde) flanking the index frame were also included in the analysis. Each frame was further divided into 4 quadrants, centred from the vessel lumen. Each quadrant was assessed for the presence of lipid, calcium, or fibrous lesion(s). A score of 1 was assigned for presence of each feature in that quadrant. (eg. Lipid: 1, calcium: 0, fibrous: 0). A total score (maximum = 4) was assigned for each feature in the frame by adding the scores from all quadrants. The average score over 11 frames (index + 10 flanking) were then calculated for each feature. A lesion was defined as “lipid-rich” if the average lipid score was the highest. Lesions which have higher fibrous or calcium scores were defined as “non-lipid” plaques (See supplemental figure 1 in Additional File 3).

**Selection of a discovery subset of subjects for proteomics analysis of plasma**

Although a stent was deployed in every PCI procedure (in this cohort), it is possible that the extent of mechanical disruption of plaques differ between individual cases as the result of different types of stent, different stent sizes, different balloon inflation time/force etc. These variations are intrinsic to the nature of clinical management and therefore unavoidable. In order to select a discovery subset of subjects for proteomics analysis, we needed a surrogate index of the “effectiveness” of plaque disruption caused by PCI in this model which is relatively independent of the downstream myocardial injury and systemic inflammation that can be observed in this experimental model.

In this regard, systemic MMP9 levels increased after plaque disruption by PCI, suggesting that it is a biological signal associated with the plaque disruption event. Further, the expression profile of MMP9 (as denoted by its AUC over the time course) did not correlate with that of troponin and CRP, suggesting that MMP9 release to be independent of the downstream myocardial injury and systemic inflammation response. We therefore concluded that MMP9 to be a surrogate index of the “effectiveness” of plaque disruption in this setting. This was the rationale of choosing the patients with the highest MMP9_AUC_ as the discovery subset for proteomics analysis of biomarker.

Similarly, the procedure of diagnostic angiography can possibly also result in inadvertent plaque injury in the patient (during the passage of guidewire etc). The same strategy therefore led us to the selection of patients with the lowest MMP9_AUC_ after diagnostic angiography as control subjects with least likelihood of plaque disruption during the experimental process.

**Proteomics methods**

**Albumin and IgG depletion from plasma samples**

Depletion of the two most abundant plasma proteins, albumin and IgG was performed in the plasma samples, using an optimised protocol.^1^ Briefly, plasma aliquots were first centrifuged at 16000g for 15min at RT to separate lipid layers. A fine pipette tip was used to aspirate 50μl from the middle layer of plasma. IgG depletion was performed by precipitation using agarose beads (Thermo Scientific ID# 20398). The beads were prewashed (1:2 volume) with 0.15mM NaCl (1:2 volume) to remove ethanol, then reconstituted with 0.15mM NaCl (1:1.5 volume). The reconstituted bead (150μL), 50μL of delipidated plasma, and 100μL of 0.15mM NaCl were transferred to a mini spin column (Costar 8161 Spin-X) and mixed thoroughly by a rotating mixer at RT for 1hr.

Subsequent steps for albumin depletion were performed in the cold room at 4°C. Plasma diluent (200μL) from the above step were transferred to a new Eppendorf tube and allowed to equilibrate to 4°C by gentle mixing on a rotating mixer for 1hr. To the 200μL of chilled plaque diluent, 158.5μL of 95% ethanol were added to achieve a final concentration of 42% ethanol. The mixture was centrifuged at 16000g x 20min at 4°C. The supernatant fraction contained albumin which remained in solution after the above procedures and was removed. The residual pellet was washed using 42% ethanol to remove any residual water, and centrifuged again at 16000g x 20min at 4°C. The pellet, containing albumin depleted protein fraction, was retrieved for the subsequent in-solution trypsin digest as described in the following section.

**Preparation of Filter Wire retained plaque debris and plasma**

The paired samples of Filter Wire and Filter Wire Control (n=2 pairs, Figure 1) were prepared concurrently. These were placed in 2ml Eppendorf tubes and washed with 500ul of MqH20. The samples were subjected to sonication in a water bath for 2 minutes to achieve cell lysis. The tissue lysate was retrieved. Both the tissue lysate and plasma samples were prepared to extract the protein fraction using the common organic extraction protocol with sequential concentration gradients of methanol, chloroform and H_2_O. This protocol utilised the variable miscibility of lipids according to the concentration of methanol in the organic solvent.^2^

# **In-solution trypsin digest for protein**

The protein pellets were processed to obtain peptide fragments using the described by Fischer et al. Briefly, 50μl of 6M urea was added to protein pellets, vortexed and sonicated for 2 minutes. Proteins were reduced with Dithiothreitol (DTT,, Sigma Aldrich), alkylated with iodoacetamide (Sigma Aldrich) and digested with Trypsin (Promega, sequencing grade, Ref V5113) overnight at 37°C in a shaker at 600rpm.

# **Desalting and purification of peptides**

Buffer A (0.1% trifluoroacetic acid) and buffer B (0.1% trifluroacetic acid with 65% acetonitrile) were prepared for the desalting step. The trypsin digested samples from section were sonicated for 1 minute, and centrifuged at 16,000g x 5min to precipitate non-peptide debris. The desalting C18 columns (Thermo Scientific SOLA tube SPH-300-010Q) were conditioned sequentially with 1ml of Buffer B (0.1% trifluroacetic acid with 65% acetonitrile) and Buffer A (0.1% trifluoroacetic acid). The solution containing peptides were loaded to the column in equal volume of Buffer A (500µl each). The column was then washed with 1ml of Buffer A. The peptides were eluted from the column with 300μL of Buffer B. The eluent was vacuum dried and stored in -80°C until analysis.

# **Data acquisition**

The desalted tryptic peptides were re-suspended in 10μL of 0.1% formic acid for LC MS/MS analysis. Each sample (0.5μL) was separated on a nanoflow reverse phase LC system (Nano Acquity, Waters) and eluted with a 2 hours linear gradient (from 3% acetonitrile plus 0.1% formic acid to 40% acetonitrile) at a flow rate of 250nl/min. The column (C18 BEH, 75μm internal diameter x 25cm length, 1.7μm particle size) was coupled to an electrospray ionisation source in positive mode. Spectra were collected from an ion trap mass analyser (LTQ-orbitrap Velos, Thermo Fisher Scientific). Peptide precursors were picked from 2 mass ranges (300-609 and 609-2,000) in technical duplicates. MS/MS was performed on the top 20 ions in each MS scan using the data-dependent acquisition mode with dynamic exclusion enabled. Fragmentation was recorded in collision induced dissociation mode in the iontrap at the collision energy of 35V with resolution 15,000 at M/Z 400. The precursor ion full scan and subsequent MS/MS spectra were acquired at a resolution of 60,000 profile mode. Poly-dimethylcyclosiloxane (m/z = 445.120025), generated in the ESI process from ambient air, was used as the reference mass for real time internal calibration throughout the experimental workflow. Technical reproducibility of the plasma samples was assessed performing principal component analysis (PCA) using proteins that are consistently detected in all plasma samples. Close clustering of the technical replicates were observed (data not shown).

MS/MS spectra were extracted from raw files and converted to MGF-format peak lists by Progenesis LC-MS^TM^ (Non-Linear Dynamics, UK, V4.0. The MGF-format peaks lists were searched with Mascot 2.3 (Matrix Science) and uploaded to Progenesis LC-MS^TM^. Only peptides identified with a false discovery rate of 1% and with ion score of >20 were accepted for further analysis.

**Supplemental Data**

In addition to MMP9, we also measured Tissue Inhibitor of Metalloproteinase 1 (TIMP1), which is another plaque protein listed in Table 1 (Row 242 in table, Uniprot accession number P01033). TIMP1 is a known inhibitor for MMP9, and has also been previously examined as a biomarker in the context of acute coronary syndrome.

We measured the level of plasma TIMP1 in the same patients described in the manuscript, and observed a significant elevation of TIMP1 at 18 hours (after PCI induced plaque disruption) in patients who presented with ACS. Whereas no significant change was observed in those who presented with SA (See Supplemental Figure 3-A in Additional File 3). This suggested that the systemic release of TIMP1 after plaque disruption in this experimental model was affected by the underlying clinical status (as compared to MMP9). When we performed the same AUC analysis to assess the correlation between TIMP1_AUC_, TnI_AUC_, and CRP_AUC_, we observed significant a correlation between TIMP1_AUC_ and TnI_AUC_ (Spearman rho=0.45, p<0.01) (Supplemental Figure 3-B in Additional File 3). Therefore, although TIMP1 was present in the plaque and significantly increased in circulation after PCI induced plaque disruption, it was not a useful index for the selection of a patient subset for discovery proteomics analysis.

**References**

1. Fischer R, Trudgian DC, Wright C, Thomas G, Bradbury LA, Brown MA, Bowness P, Kessler BM. Discovery of candidate serum proteomic and metabolomic biomarkers in ankylosing spondylitis. Mol Cell Proteomics 2012;**11**(2):M111 013904.

2. Bligh EG, Dyer WJ. A rapid method of total lipid extraction and purification. Can J Biochem Physiol 1959;**37**(8):911-7.
