# Supplementary material for: A novel workflow combining plaque imaging, plaque and plasma proteomics identifies biomarkers of human coronary atherosclerotic plaque disruption
Source: Clin Proteomics. 2017 Jun 19;14:22. doi: 10.1186/s12014-017-9157-x (PMC5477097; doi:10.1186/s12014-017-9157-x)

#### Supplemental Figure 1: Characterisation of plaque morphology by optical coherence tomography. For each culprit lesion, the image frame containing the minimal luminal area (MLA) was identified and selected as the index frame. Ten additional frames (5 antegrade + 5 retrograde) flanking the index frame were also included in the analysis. Each frame was further divided into 4 quadrants, centred from the vessel lumen. Each quadrant was assessed for the presence of lipid, calcium, or fibrous lesion(s). A score of 1 was assigned for presence of each feature in that quadrant. (eg. Lipid: 1, calcium: 0, fibrous: 0). A total score (maximum = 4) was assigned for each feature in the frame by adding the scores from all quadrants. The average score over 11 frames (index + 10 flanking) were then calculated for each feature. A lesion was defined as “lipid-rich” if the average lipid score was the highest. Lesions which have higher fibrous or calcium scores were defined as “non-lipid-rich” plaques.

**Supplemental Figure 2:** **Acute elevation of systemic lipopolysaccharide binding protein after plaque disruption.** We identified lipopolysaccharide binding protein (LBP) as a protein that is present in human coronary artery plaque and is significantly elevated in plasma immediately after plaque disruption. The level of plasma LBP was measured in individual plasma samples used for the proteomics analysis (n=10 in each group). Significant elevantion of plasma LBP level was observed in patients who underwent PCI induced plaque disruption (1.44 times increase [95% confidence interval of 1.07 to 1.81]) but remained unchanged in the control group who underwent diagnostic angiography without plaque disruption. * denotes p<0.05. Error bars represent standard error of means.

**Supplemental Figure 3: Systemic elevation of TIMP1 after plaque disruption was observed in patients who presented with acute coronary syndrome (ACS), and correlated with the extent of myocardial damage**. **Panel A**: The levels of plasma Tissue Inhibitor of Tissue Inhibitor of Metalloproteniase 1 (TIMP1) in this study cohort was measured using bead based immuno-assay as described in the methods. Systemic elevation of TIMP1 was observed at 18 hours after stenting induced plaque disruption in patients who presented with ACS, but not in patients who presented with stable angina (SA). (data presented as mean and SEM, * p<0.05) **Panel B**: The area under curve (AUC) for TIMP1 over the time course was calculated as described in the methods section. There was significant correlation between TIMPAUC and the TnIAUC over the time course, suggesting that the TIMP1 changes observed after stent induced plaque disruption may be confounded by the downstream myocardial damage after coronary artery stenting.


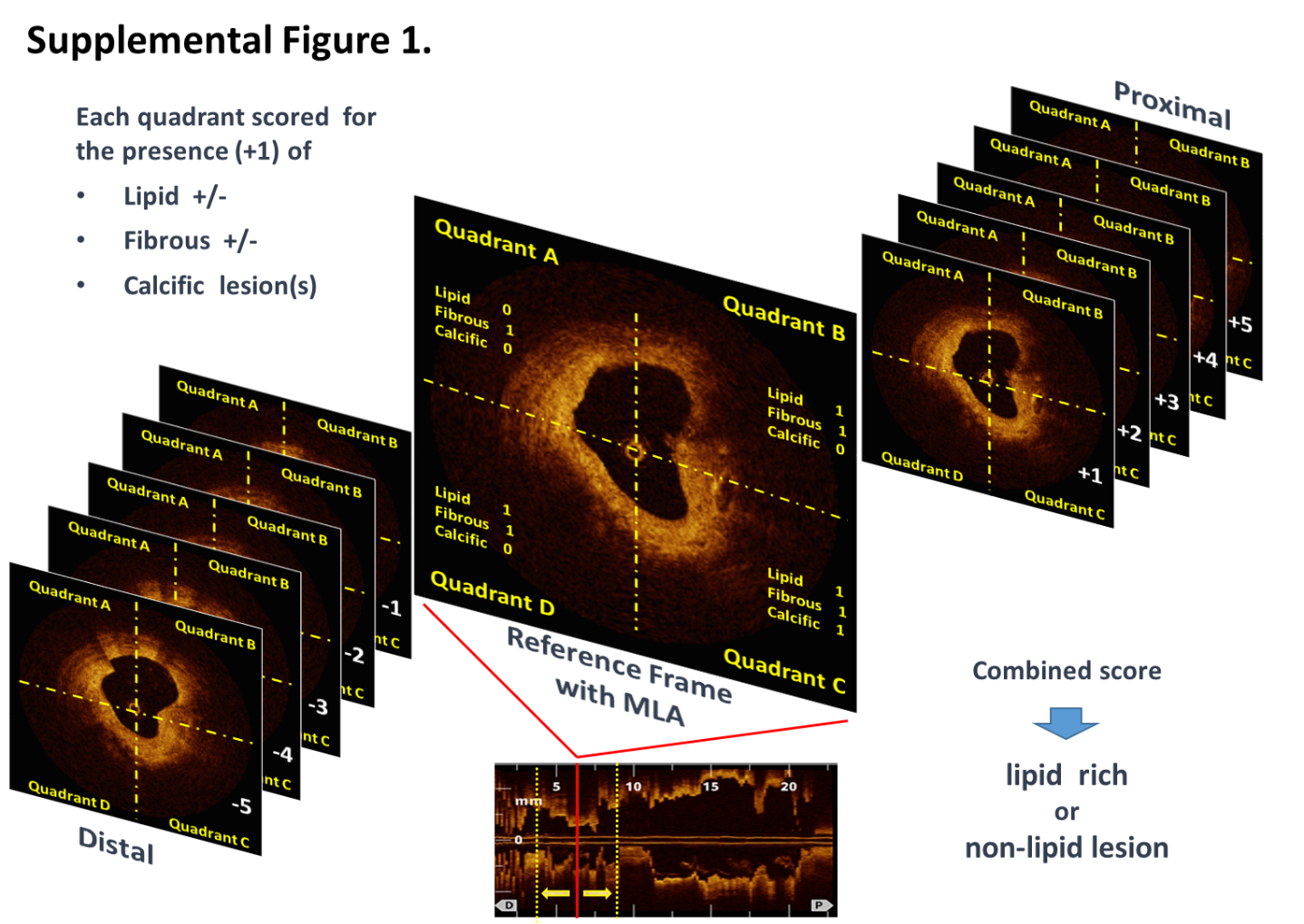


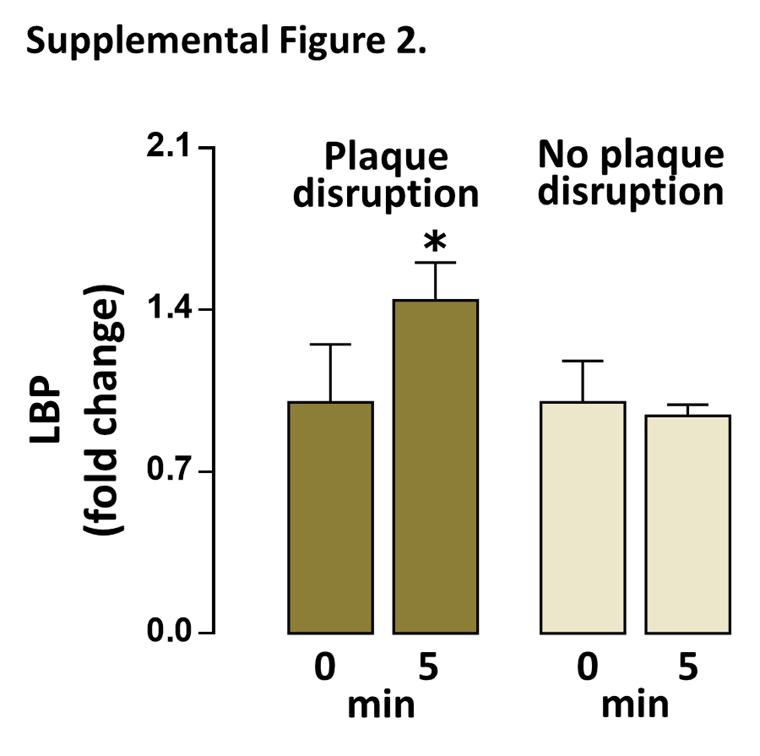


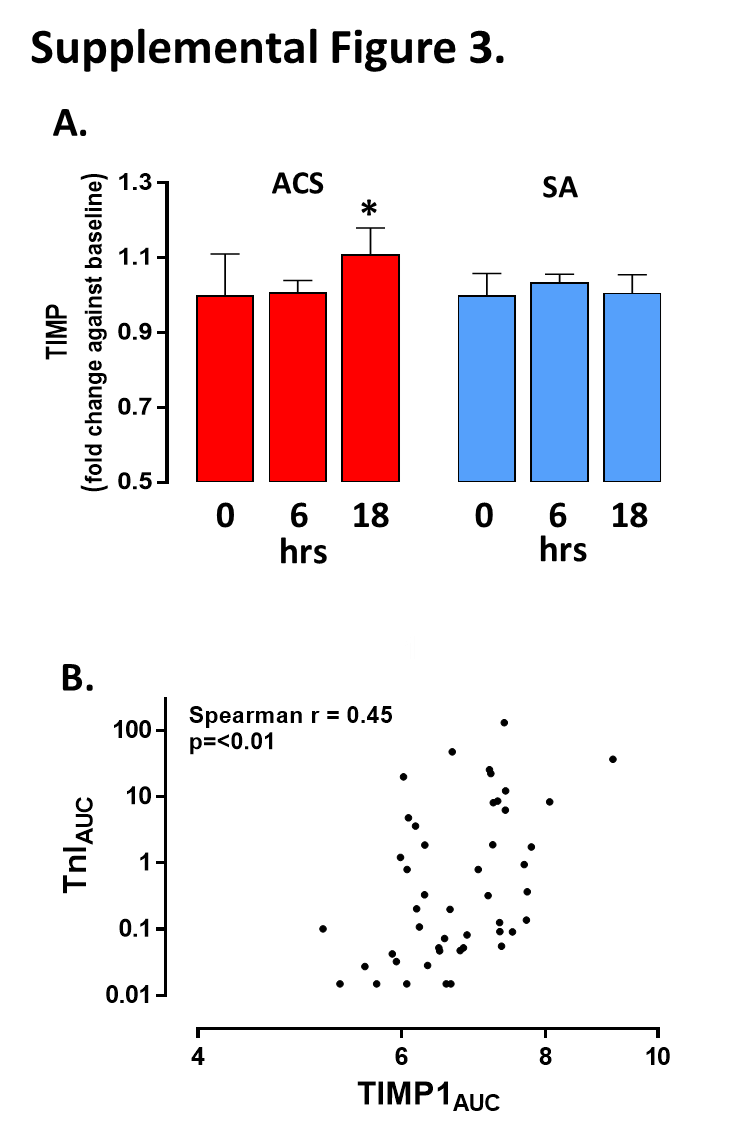

Supplement: Supplementary file 3 — Additional file 3. Changes in lipopolysaccharide binding protein after plaque disruption. [file 12014_2017_9157_MOESM3_ESM.docx]
